# Influence of Medicaments on the Heart

**Published:** 1869-04

**Authors:** 


INFLUENCE OF MEDICAMENTS ON THE HEART.—The *Proceedings of the French Society of Therapeutics* contains a paper on this point by M. Bordier, who recommends that in all therapeutical experiments the sphygmograph should be employed. By the use of this instrument, M. Bordier has been able readily to distinguish between drugs which increase and those which diminish the tension of the vessels.

